# Analysis of serum amino acids and tryptophan metabolites to predict hepatic encephalopathy in portal hypertension patients receiving a transjugular intrahepatic portal shunt (TIPS)

**DOI:** 10.3389/fphar.2025.1546665

**Published:** 2025-08-28

**Authors:** Li Bao, Yifan Lv, Chunjing Yang, Jingfeng Li, Fuquan Liu, Zhengyuan Shi

**Affiliations:** ^1^ Department of Pharmacy, Beijing Shijitan Hospital, Capital Medical University, Beijing, China; ^2^ Beijing Key Laboratory of Bio-characteristic Profiling for Evaluation of Rational Drug Use, Beijing, China; ^3^ Department of Interventional Therapy, Beijing Shijitan Hospital, Capital Medical University, Beijing, China

**Keywords:** hepatic encephalopathy, portal hypertension, transjugular intrahepatic portal shunt, amino acids, tryptophan metabolites

## Abstract

**Introduction:**

The incidence of hepatic encephalopathy (HE) after transjugular intrahepatic portal shunt (TIPS) is over 30%, how to decrease the occurrence rate of HE is one of the most challenging issues after TIPS surgery. The aim of this study is to identify the potential biomarker that can predict the occurence of HE after TIPS in patients with portal hypertension (PH), and provide the theoretical support for early clinical diagnosis and prevention.

**Methods:**

A total of 50 patients with PH underwent TIPS were enrolled with completed clinical information in this study, and they were divided into two groups with or without the complications of HE.

**Results:**

We quantitatively determined 20 serum amino acids, and found that 4 amino acid levels were increased significantly in patients who complicated HE after TIPS. Screened by the specific screening criteria (VIP > 1.0; P < 0.05; FC > 1.5, or FC < 0.67) on MetaboAnalyst 5.0 on-line platform, tryptophan (TRP) were identified as the potential biomarker (50.31 μmol/L in non-HE group vs 100.21 μmol/L in HE group). Subsequently the detection of serum tryptophan metabolites revealed that the higher levels of kynurenine (KYN) and 2,3-Pyridinedicarboxylic acid (QA) were observed in the patients complicated HE after TIPS. ROC multivariate analysis indicated that the combination of TRP, KYN and QA provied the better predictive value for HE occurrence in PH patients underwent TIPS (AUC = 0.92).

**Discussion:**

This study provided the theoretical suggestion to the clinical doctor which the level of serum TRP/KYN/QA of patients should be detected before TIPS, and It is of great significance for patient prognosis and reducing the occurrence of HE after TIPS.

## 1 Introduction

Portal hypertension (PH) is a common clinical syndrome accompanied with ascites, varices, and bleeding, and it is the leading cause of death in patients with chronic liver disease (Simonetto et al., 2019). Currently, the most effective treatment method is transjugular intrahepatic portal shunt (TIPS) (Horhat et al., 2021). However, TIPS reduces the blood flow, leading to insufficient ammonia clearance and elevated blood ammonia levels; the excess ammonia enters the central nervous system through the blood–brain barrier and further impairs liver function, leading to postoperative complications such as hepatic encephalopathy (HE).

At present, the incidence of HE after TIPS exceeds 30% (Zuo et al., 2019), and the majority of patients show improvement with the standard treatment, but 3%–7% of patients develop refractory HE (Pereira et al., 2016; Nardelli et al., 2023). Therefore, the occurrence of HE is one of the most challenging issues after TIPS surgery. Various strategies to decrease HE incidence have been explored, including selective puncture of the left branch of the portal vein during TIPS (Luo et al., 2019) and reducing stent diameter (Madoff et al., 2004), although the latter may increase the risk of portal hypertension-related complications such as variceal bleeding and refractory ascites. Other approaches include early low-protein diets to reduce blood ammonia levels and prophylactic use of lactulose, rifaximin, and L-ornithine L-aspartate (LOLA) to enhance ammonia clearance (Luo et al., 2016; Riggio et al., 2005; American Association for the Study of Liver Diseases, 2014). However, these methods are not universally effective, and there remains a need for reliable models or strategies to predict and prevent HE after TIPS. There are still many retrospective analyses indicating that the history of preoperative HE, bilirubin level, Child–Pugh and the model of end-of-stage liver disease (MELD) scores, international normalized ratio (INR) indicators, and the usage of PPI are all related to the severity and frequency of postoperative HE (Gangwani et al., 2024; Fonio et al., 2017). However, there are no confirmed models or strategies to predict or intervene in the occurrence of HE after TIPS, but we can see that the clinicians and related researchers all over the world are working hard for them.

In our previous research on untargeted metabolomics, we found that there were significantly changed metabolites in the peripheral and portal serum after TIPS, which may be closely related to early changes in liver function after TIPS. In addition, in the pathway analysis, both elevated and depleted metabolites were mainly enriched in amino acid metabolism (Chen et al., 2023); therefore, we quantified the changes in serum amino acids in this study. The amino acid hypothesis of HE made significant progress in clinical and biochemical research works in the 1970s and 1980s, especially the treatment of branched-chain amino acid (BCAA) supplementation that can improve nitrogen balance and reduce the recurrence of HE in patients with cirrhosis (Horst et al., 1984), which has received widespread attention. However, some studies have shown that BCAA supplementation has few effects on occult HE (Gluud et al., 2017). In our experiment, we found a significant difference in tryptophan (TRP) levels between the two groups, while BCAAs did not show a significant difference. Further analysis of TRP metabolites revealed that the levels of kynurenine (KYN) and 2,3-pyridinedicarboxylic acid (QA) in the serum of patients with PH complicated with HE after TIPS surgery were significantly higher than those without HE complications. We speculate that the occurrence of HE may be related to the KYN/IDO (indoleamine-2,3-dioxygenase) pathway of TRP metabolism. Our research might provide a theoretical basis for prevention to the occurrence of HE after TIPS in PH patients and improve the patients’ prognosis and quality of life.

## 2 Materials and methods

### 2.1 Reagents

UPLC-MS-grade methanol, acetonitrile, and isopropanol were obtained from Thermo Fisher Scientific (Fair Lawn, NJ, United States). UPLC-MS-grade formic acid was purchased from Sigma-Aldrich (St. Luis, MO, United States). Ultrapure water (18.2 MΩ) was produced using a Milli-Q water purification system (Millipore, Bedford, MA, United States). Amino acids, tryptophan metabolite standards, and ketotifen were purchased from Sigma-Aldrich (Gillingham, United Kingdom). Isotopically labeled amino acids for use as internal standards (ISs) were obtained from Cambridge Isotope Laboratories (MA, U.S.A.) or QMX Laboratories (Essex, United Kingdom), and the AccQTag Ultra reagent kit was obtained from Waters Corporation (Milford, MA, United States).

### 2.2 Human serum sample collection and preparation

Fifty patients with PH who underwent TIPS were enrolled in our experiment, of whom 25 patients faced complications of HE after TIPS. Inclusion criteria of the patients were as follows: 1) 18–80 years old, 2) completed clinical information, and 3) no other metabolic diseases such as diabetes. All participants were from Beijing Shijitan Hospital, Capital Medical University (Beijing, China), who provided written informed consent, and the study was approved by the Ethics Committee of Beijing Shijitan Hospital Affiliated to Capital Medical University (2019(01)) and conducted under the guidelines of the Helsinki Declaration.

The jugular vein blood samples were collected before TIPS was performed, and the blood samples were centrifuged at 3,500 rpm for 10 min at 4°C to obtain the serum, which was stored in a refrigerator at −80°C for the following analysis.

### 2.3 The analysis of amino acids

#### 2.3.1 The preparation of standards

The amino acid standards were dissolved in a 1 M stocked solution and diluted with acetonitrile–water solution (1:1) to a mixed standard concentration of 1, 2, 4, 10, 20, 40, 100, 200, and 400 μM. The Cambridge isotope internal standard was prepared to the concentration of 10 μg/mL with acetonitrile–water solution (1:1) (Shi et al., 2023; Bao et al., 2024).

#### 2.3.2 Sample pretreatment method

Before analysis, serum samples were thawed at 4°C. Then, 10 μL of thawed serum, 10 μL of water, and 5 μL of the internal standard (10 μg/mL) were mixed and vortexed for 5s; 40 μL of isopropanol was added and incubated at 4°C for 10 min to precipitate the protein. After centrifugation at 12,000 rpm for 15 min, 10 μL of the supernatants was placed in another Eppendorf tube for derivatization.

The preparation of the derivatization reagent followed the instructions of the AccQTag Ultra reagent kit: 1 mL acetonitrile was added to the AccQTag Ultra reagent powder, vortexed for 5 s, and dissolved by heating at 55°C for 10 min to obtain the derivatization reagent. Then, 70 μL of borate buffer (pH 8.6) and 20 μL of the prepared derivatization reagent were added into the 10-μL sample supernatant, vortexed for 10 s, and then heated at 55°C for 10 min. After the completion of derivatization, the sample was diluted with 900 μL of water for UPLC-MS analysis.

#### 2.3.3 Chromatographic and mass spectrometric conditions

Chromatography was carried out using an ACQUITY UPLC HSS T3 column (150 mm × 2.1 mm, 1.8 μm) (Waters, United States), with the following conditions: the column temperature was set as 55°C; the injection temperature was 4°C; the injection volume was 2 μL, and the flow rate was 0.6 mL/min. Mobile phase A: 0.1% (v/v) formic acid in H2O, mobile phase B: 0.1% (v/v) formic acid in acetonitrile; gradient elution procedure: 0–0.5 min, keep 4% B for 0.5 min; 0.5–2.5 min, from 4% B to 10% B; 2.5–5.0 min, from 10% B to 28% B; 5.0–5.1 min, from 28% B to 95% B; 5.1–6.1 min, keep 95% B for 1 min; 6.1–6.2 min, return to 4% B; 6.2–7.5 min, keep 4% B to equilibrate the column.

Mass spectrometric conditions were as follows: UPLC-MS detection was carried out via electrospray ionization (ESI) in the positive ion mode using multiple reaction monitoring (MRM) for the quantification of each compound. The capillary voltage was set as 1.5 kV; source offset was set as 50 V; desolvation temperature was set as 600°C; source temperature was set as 150°C; desolvation gas flow was set as 1000 L/h; cone gas flow was set as 150 L/h; nebulizer gas was set as 7.0 bar; collision gas was set as 0.15 mL/min. The MS parameters including cone energy (CE) and declustering potential (DP) and the standard curve were described in our previous paper (Bao et al., 2024).

### 2.4 The analysis of TRP metabolites

#### 2.4.1 The preparation of standards

TRP metabolite standards were dissolved in methanol with the concentration of 1 mg/mL and stocked under −80°C, and standard samples for the calibration curve were prepared, prior to use, as a mixture solution, considering each range of measurement concentrations. In addition, Ketotifen was used as an internal standard for TRP metabolite detection (Takada et al., 2018).

#### 2.4.2 Sample pretreatment method

For the TRP metabolite assay, 10 μL of the internal standard was added to the 40-μL serum sample, and then, the mixture was diluted to 200 μL with the methanol solution containing 0.1% formic acid (v/v), vortexed, and incubated to precipitate the protein at −20°C for 1 h. After centrifugation at 12,000 rpm for 20 min at 4°C, the supernatant was taken and dried using a concentrator vacuum centrifuge (Jiaimu, China). Finally, the residue was redissolved in 200 μL of methanol solution containing 0.1% formic acid for UPLC-MS detection.

#### 2.4.3 Chromatographic and mass spectrometric conditions

TRP metabolite analysis was performed using the UPLC-MS method in the positive mode; an AB SCIEX Triple TOF 5500 mass analyzer in positive ion mode, combined with the Waters ACQUITY UPLC system, was used.

Chromatographic conditions: The TRP metabolites were separated through reverse-phase chromatography using an ACQUITY UPLC CSH C18 column (2.1 mm × 100 mm, 1.7 μm) (Waters, United States) with a gradient elution. Mobile phases were 0.1% formic acid solution (A) and acetonitrile (B) with the gradient elution; acetonitrile (B) was started from 5%, and then from 5% to 95% concentration in 10 min and kept for 1 min; the flow rate was set as 0.4 mL/min. The temperature of the column was 40°C.

Mass spectrometric conditions were as follows: the capillary voltage was set as 1.5 kV; the source offset was set as 50 V; the desolvation temperature was maintained at 600°C, with a cone gas flow of 150 L/h; the source temperature was 150°C; the desolvation gas flow was 1000 L/h; the nebulizer gas was set as 7.0 bar; the collision gas was 0.15 mL/min. The MS parameters, including precursor ion, product ion, CE, and DP, are shown in Table 3.

### 2.5 Data analysis

The raw data were collected using a Waters Masslynx data processing workstation (Waters Co., Ltd.), and the concentrations of amino acids and tryptophan metabolite were then calculated following the standard curve. The difference in the clinical parameters and the target substance concentration between two groups was compared using the univariate t-test with GraphPad Prism 9.5 software (GraphPad Software, United States). Amino acids with variable importance in the projection (VIP) > 1, P < 0.05, and fold change (FC) ≥1.5 or ≤0.67 were considered to have significant differences by using the MetaboAnalyst 5.0 online platform and shown in volcano and heatmap diagrams. Receiver operating characteristic curve (ROC) analysis was performed on the MetaboAnalyst 5.0 online platform with biomarker analysis function to calculate the predictive value of the metabolites and diseases.

## 3 Results

### 3.1 Study population

A total of 50 patients with PH who underwent TIPS were enrolled in this study, and they were divided into two groups: HE group (25 of whom had complications of HE after TIPS) and non-HE group (the other 25 of whom did not have complications of HE after TIPS). The clinical parameters including age, body mass index (BMI), hepatic venous pressure gradient (HVPG), portal pressure gradient (PPG), alanine transaminase (ALT), aspartate transaminase (AST), albumin, total bilirubin (TBIL), indirect bilirubin (IBIL), direct bilirubin (DBIL), international normalized ratio (INR), ascites, Child–Pugh score, variceal bleeding, and West Haven category were recorded. At 1 year after TIPS surgery, there were three deaths (one cerebral hemorrhage and two cases of liver failure) in the HE group and one death (liver cancer) in the non-HE group. The West Haven criteria were used to quantify and define HE (Blei and Cordobal, 2001). There were no statistically significant differences in the recorded clinical parameters between two groups (except for albumin, P = 0.02), and detailed characteristics are provided in Table 1.

**TABLE 1:** Baseline characteristics of patients.

	Non-HE	HE
Gender (% males)	72% (18/25)	76% (19/25)
Age (years)	51.88 ± 2.71	56.84 ± 2.64
BMI	23.07 ± 0.45	21.43 ± 0.70
Cause of PH:		
Alcoholic	1	2
Virus	15	17
Budd-Chiari syndrome	2	0
Idiopathic portal hypertension	2	0
Autoimmune liver disease	0	0
Other	5	6
HVPG (mmHg)	18.94 ± 1.92	17.28 ± 1.16
PPG (mmHg)	25.65 ± 1.46	23.76 ± 1.39
ALT (U/L)	32.68 ± 7.03	38.54 ± 5.43
AST (U/L)	37.36 ± 16.09	58.04 ± 23.07
Creatinine (μmol/L)	65.60 ± 4.64	71.33 ± 7.23
Albumin (g/L)	37.34 ± 1.05	34.15 ± 0.82*
TBIL	28.24 ± 3.85	30.41 ± 3.55
IBIL	13.55 ± 1.73	15.32 ± 5.91
DBIL	12.00 ± 2.32	13.25 ± 1.86
INR	1.34 ± 0.05	1.32 ± 0.04
Cancer	20% (5/25)	20% (5/25)
NH3	49.04 ± 3.47	54.32 ± 4.59
Stent diameter	8mm: 23	8mm: 20
	10mm: 2	10mm: 5
Ascites	Absent: 14	Absent: 9
	Mild: 7	Mild: 12
	Severe: 4	Severe: 4
Child-Pugh score	6.58 ± 0.39	7.05 ± 0.27
Child-Pugh category	A: 14	A: 8
	B: 8	B: 14
	C: 2	C: 1
	ND: 1	ND: 2
Variceal bleeding	Absent: 5	Absent: 4
	Present: 20	Present: 21
West-haven category	--	I: 7
		II: 5
		III: 4
		IV: 9

All data were presented as mean ± SEM; *P < 0.05 compared with the non-HE group.

### 3.2 Targeted analysis of serum amino acids in PH patients who underwent TIPS

We analyzed the serum amino acid concentration of PH patients with/without HE after TIPS using the derivatization method with 6-aminoquinolyl-n-hydroxysuccinimidyl carbamate, and we can find the UPLC-MS spectra in Supplementary Figure S1. Here, we quantitatively analyzed 20 amino acids including 8 essential amino acids and 12 other amino acids that play an important role in protein synthesis. The concentrations of 20 amino acids were quantitatively determined according to the linear equation. The MS parameters, standard curves, and the data from healthy controls have been previously published (Bao et al., 2024).

**FIGURE 1:**
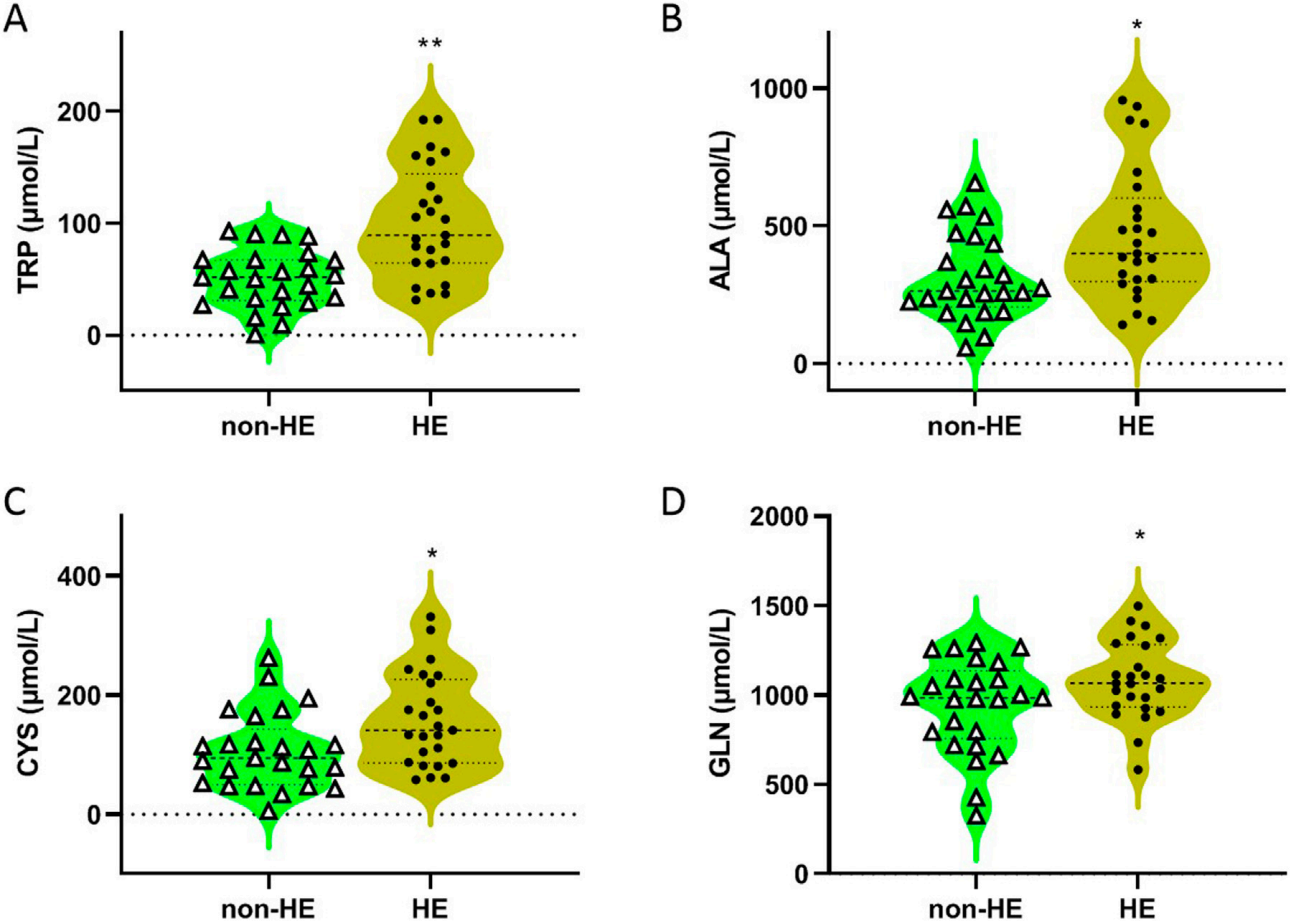
Concentrations of TRP **(A)**, ALA **(B)**, CYS **(C)**, and GLN **(D)** in serum of PH patients with/without HE after TIPS. *P < 0.05 and **P < 0.01 compared with the non-HE group.

As shown in Table 2 and Figure 1, four amino acids (TRP, ALA, CYS, and GLN) exhibited significant differences between two groups (50.31 μmol/L in the non-HE group vs. 100.21 μmol/L in the HE group, 323.05 μmol/L vs. 464.44 μmol/L, 106.61 μmol/L vs. 158.03 μmol/L, and 943.40 μmol/L vs. 1,084.82 μmol/L, respectively), and levels of these four amino acids were significantly higher in the HE group than in the non HE group, which is consistent with our previous research findings that the occurrence of HE after TIPS surgery is related to amino acid metabolism (Chen et al., 2023).

**TABLE 2:** Amino acid concentrations in serum of PH patients with/without HE after TIPS.

	Amino acid	Abbreviation	Non-HE (μmol/L)	HE (μmol/L)
1	Threonine	THR	375.09 ± 37.49	420.17 ± 45.56
2	Leucine	LEU	30.31 ± 3.28	35.20 ± 2.93
3	Isoleucine	ILE	80.41 ± 8.87	88.18 ± 9.62
4	Valine	VAL	117.07 ± 11.23	136.19 ± 10.95
5	Phenylalanine	PHE	61.27 ± 7.46	77.76 ± 5.56
6	Methionine	MET	81.65 ± 32.11	71.10 ± 5.38
7	Tryptophan	TRP	50.31 ± 7.10	100.21 ± 11.95**
8	Lysine	LYS	151.75 ± 16.04	200.95 ± 26.67
9	Glycine	GLY	263.87 ± 38.72	353.50 ± 41.58
10	Tyrosine	TYR	58.32 ± 7.15	71.08 ± 5.38
11	Histidine	HIS	120.18 ± 14.02	169.62 ± 23.02
12	Alanine	ALA	323.05 ± 35.56	464.44 ± 50.75*
13	Cysteine	CYS	106.61 ± 12.75	158.03 ± 15.58*
14	Aspartic acid	ASP	12.98 ± 2.77	20.23 ± 6.61
15	Proline	PRO	188.88 ± 31.25	237.31 ± 26.24
16	Serine	SER	163.53 ± 21.37	185.63 ± 19.97
17	Glutamine	GLU	28.60 ± 6.33	46.87 ± 7.29
18	Arginine	ARG	122.54 ± 15.31	156.58 ± 14.32
19	Asparagine	ASN	69.08 ± 9.82	83.53 ± 9.00
20	Glutamine	GLN	943.40 ± 51.80	1084.82 ± 43.37*

All data were presented as mean ± SEM; *P < 0.05 and **P < 0.01 compared with the non-HE group.

### 3.3 Screening and identification of differential amino acids

**FIGURE 2:**
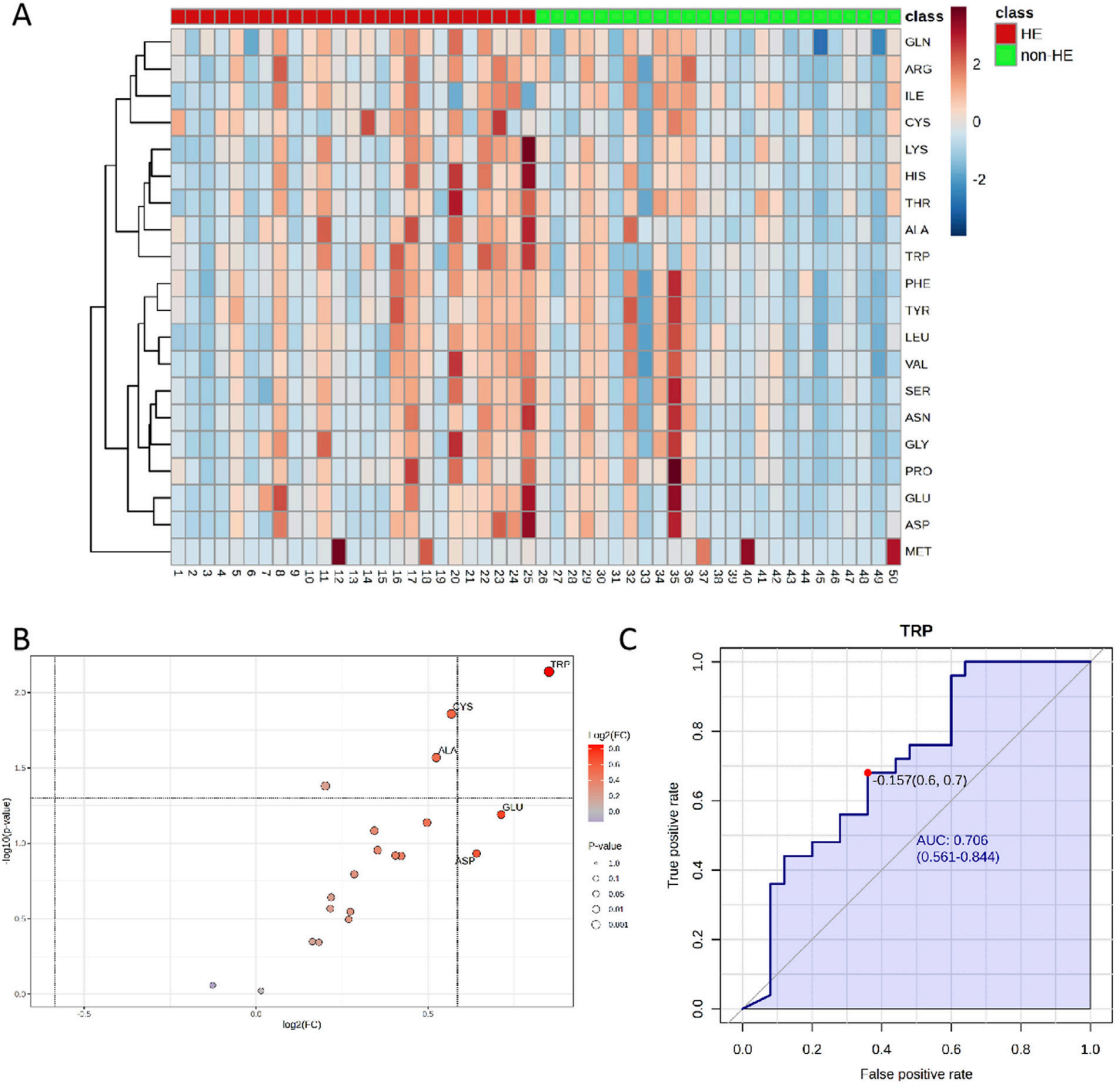
**(A)** Heatmaps of normalized amino acid concentrations in the serum sample. Columns represent the samples (post-TIPS patients with/without HE), and rows represent amino acids. **(B)** The volcano analysis of the significantly changes in amino acid levels in PH patients with HE after TIPS (FC was set as >1.5 or <0.67, and P < 0.05) and **(C)** the ROC curve of TRP. The blue line represents the 95% CI (AUC>0.7; P < 0.05).

The UPLC-MS raw data were imported into the Metaboanalyst 5.0 online platform for further analysis. As shown in the heatmap diagram, hierarchical clustering analysis (HCA) results of the 20 amino acids (left of the heat map) are displayed (Figure 2A), and 20 amino acids in the two groups were significantly distinguished, which indicated that there were differences in the composition and concentration of amino acids. Then, we obtained the VIP (Supplementary Figure S2) and FC values (Supplementary Figure S3) that were used to identify differential amino acids; according to the specific screening criteria (VIP>1.0; P < 0.05; FC > 1.5 or FC < 0.67), we found that TRP had the greatest difference in the volcano diagram between these two groups (Figure 2B). Univariate ROC analysis revealed that TRP was related to the occurrence of HE after TIPS in PH patients with the AUC of 0.706 (Figure 2C).

### 3.4 Targeted analysis of TRP metabolites in PH patients who underwent TIPS

TRP is an essential amino acid that serves as a precursor for the synthesis of multiple important bioactive compounds. The TRP levels in the human body depend on the dietary intake and TRP metabolic pathways, including the serotonin pathway, the kynurenine/IDO (indoleamine-2,3-dioxygenase) pathway, and the indole/AHR (aryl hydrocarbon receptor) pathway. Therefore, we will further identify TRP metabolites involved in these three pathways. Here, we quantified 13 TRP metabolites using the UPLC-MS method (Supplementary Figure S4; Supplementary Table S1).

**FIGURE 3:**
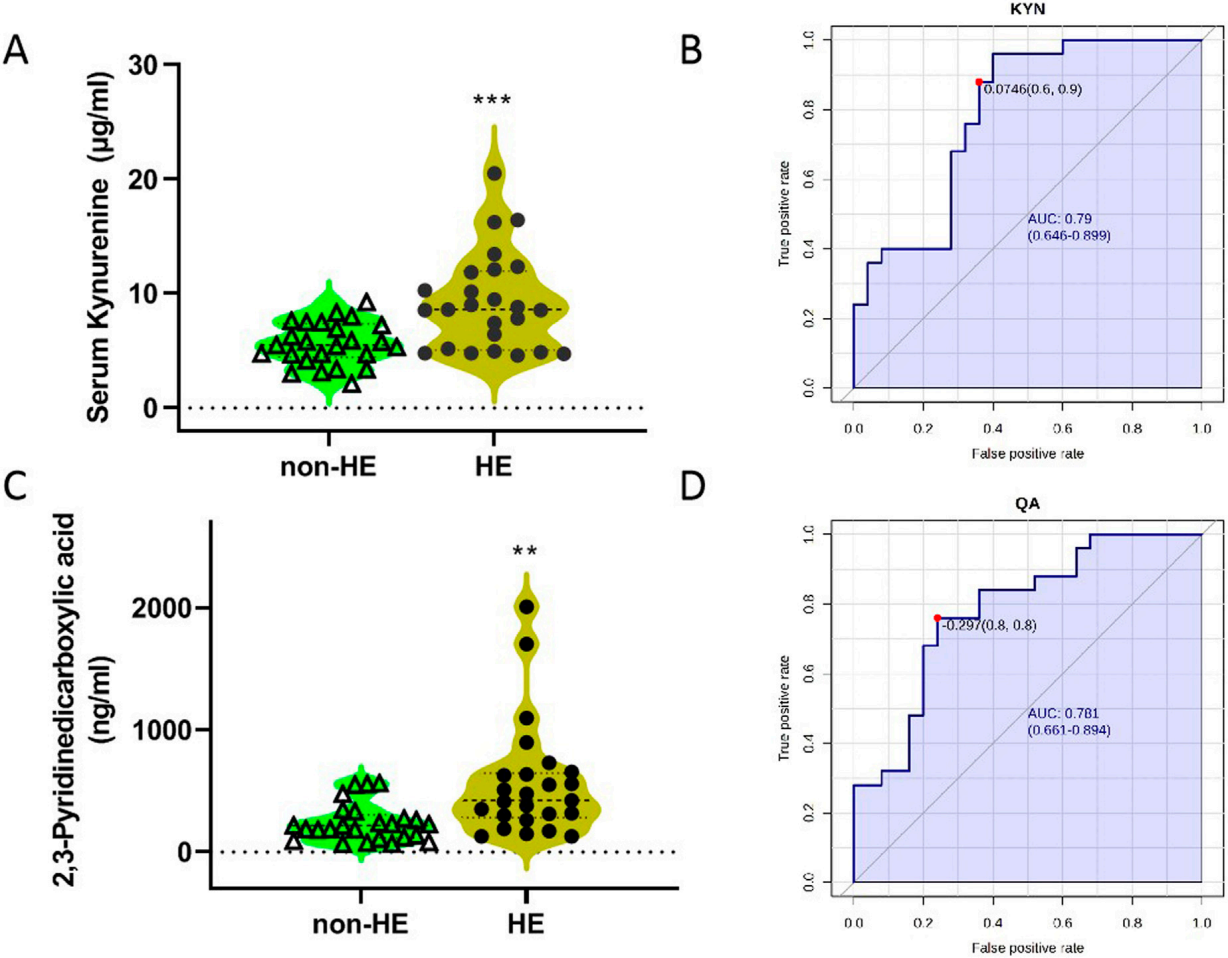
Concentrations of KYN **(A)** and QA **(C)** in two groups, and the ROC curves of KYN **(B)** and QA **(D)**. The blue line represents the 95% CI.

Analyses of serum TRP metabolites revealed that TRP, 3-indole-lactic acid (ILA), KYN, and xanthurenic acid (XA) were the most abundant serum TRP metabolites, with the concentration more than 1 μg/mL; however, N-acetyl-5-hydroxytryptamine (NAM) and melatonin (MT) were not detected under our testing conditions due to its low content (Table 3). Furthermore, we observed that there were significant differences in TRP, kynurenine, and 2,3-pyridinedicarboxylic acid (quinolinic acid, QA) between the two groups (Table 3; Figures 3A,C); these findings suggested that the occurrence of HE after TIPS may be related to the kynurenine/IDO pathway.

**TABLE 3:** TRP metabolite concentrations in serum of PH patients with/without HE after TIPS.

	Amino acid	Abbreviation	Molecular formula	Precursor ion (m/z)	Product ion (m/z)	DP^a^	CE^b^	HE group	Non-HE group
1	Tryptophan	TRP	C_11_H_12_N_2_O_2_	205.1	188.1	74	16	22.40 ± 0.96 μg/ml	14.30 ± 0.73 μg/ml*
2	Serotonin	5-HT	C_10_H_12_N_2_O	177.1	160.1	52	14	20.80 ± 4.03 ng/ml	29.86 ± 6.89 ng/ml
3	5-hydroxytryptophan	5-HTP	C_11_H_12_N_2_O_3_	221.1	162.1	69	24	16.82 ± 3.07 ng/ml	6.03 ± 1.34 ng/ml
4	N-acetyl-5-hydroxytryptamine	NAM	C_12_H_14_N_2_O_2_	219.1	160.0	138	20	--	--
5	Melatonin	MT	C_13_H_16_N_2_O_2_	233.1	174.0	147	22	--	--
6	5-hydroxyindole-3-acetic acid	HIAA	C_10_H_9_NO_3_	192.1	146.1	125	22	27.45 ± 3.28 ng/ml	20.16 ± 2.40 ng/ml
7	3-Indoleacetic acid	IAA	C_10_H_9_NO_2_	176.1	130.1	119	22	366.55 ± 40.63 ng/ml	328.22 ± 38.53 ng/ml
8	3-Indole-lactic acid	ILA	C_11_H_11_NO_3_	206.1	117.9	119	29	3.85 ± 0.44 μg/ml	3.19 ± 0.64 μg/ml
9	Indolepropionic acid	IPA	C_11_H_11_NO_2_	190.0	130.0	106	24	62.60 ± 19.98 ng/ml	51.35 ± 17.39 ng/ml
10	Indole-3-acetamide	IAM	C_10_H_10_N_2_O	175.1	130.0	60	20	2.09 ± 0.85 ng/ml	1.12 ± 0.52 ng/ml
11	Kynurenine	KYN	C_10_H_12_N_2_O_3_	209.3	93.9	75	18	9.29 ± 0.84 μg/ml	5.63 ± 0.37 μg/ml*
12	2,3-Pyridinedicarboxylic acid	QA	C_7_H_5_NO_4_	167.9	150.0	103	13	558.44 ± 92.16 ng/ml	238.83 ± 30.80 ng/ml*
13	Xanthurenic acid	XA	C_10_H_7_NO_4_	206.0	159.9	132	26	1.83 ± 0.18 μg/ml	1.61 ± 0.17 μg/ml

All data were presented as mean ± SEM; *P < 0.05 compared with the non-HE group.

^a^ DP, declustering potential.

^b^ CE, cone energy.

Then, we imported the data to the biomarker analysis module on the Metaboanalyst 5.0 online platform to obtain the univariate ROC curve analysis for individual biomarkers, as shown in Figures 3B,D. The AUC values of kynurenine and 2,3-pyridinedicarboxylic acid were 0.790 and 0.781, respectively (Supplementary Table S1). When the optimal cutoff score for the KYN model is 0.0764, the sensitivity is 60% and the specificity is 90%. In addition, we imported these data for multivariate analysis; these three metabolites as a combined form had an AUC of 0.92, indicating that they had the better predictive value in predicting whether HE occurs in PH patients who underwent TIPS (Supplementary Table S1).

## 4 Discussion

The primary pathological mechanism of HE is elevated blood ammonia levels; clinicians in various countries are striving to prevent the occurrence of HE by reducing the body’s ammonia content. The most commonly used method is L-ornithine L-aspartate (LOLA), which optimizes liver metabolic pathways to eliminate blood ammonia, thereby reducing the occurrence of HE (American Association for the Study of Liver Diseases, 2014). In a double-blind randomized controlled trial, 73 volunteers receiving LOLA treatment exhibited a significantly lower HE recurrence rate of 12.3% compared to the placebo group, which has an HE recurrence rate of 27.7% (Varakanahalli et al., 2018). Another study focusing on high-grade HE patients undergoing online hemodiafiltration revealed that tyrosine and phenylalanine were effectively removed through the hemodiafiltration, and the serum NH3 level was decreased, which contributed to an improvement in the level of consciousness (Sagata et al., 2024). However, these approaches are remedial measures taken after the occurrence of HE, and can we make predictions before HE to prevent its occurrence?

In our previous research, we found significant early changes in metabolites (peptides, amino acids, and lipid metabolites) in the peripheral and portal serum of PH patients after TIPS. Of note, the enrichment analysis showed that the increased metabolites were mainly enriched in amino acid metabolism pathways, indicating that amino acids might be related to metabolite changes after TIPS (Chen et al., 2023). Based on these findings, the current study utilized preoperative jugular vein blood and conducted a 1-year follow-up on these patients (most patients’ HE occurs within 3 months after TIPS); finally, 25 patients with complicated HE within 3 months after TIPS were enrolled in our research, with the aim of predicting the occurrence of subsequent HE through preoperative serum metabolites.

We specifically targeted the detection of 20 serum amino acids to observe the changes after TIPS in PH patients and found that four of them (TRP, ALA, CYS, and GLN) were significantly increased in PH patients after TIPS complicated with HE. The urea cycle is a primary mechanism for ammonia clearance in the human body; when the urea cycle was damaged due to liver disease, the ability for ammonia clearance also decreased. Consequently, the glutamine synthesis pathway compensatively increased the ability of clearing excess ammonia from the blood (Katayama and Kakita, 2024), which can explain the increased GLN levels observed in the HE group. Combined with the multivariate and univariate statistical analyses on the Metaboanalyst 5.0 platform, we set the specific screening criteria (VIP>1.0; P < 0.05; FC > 1.5 or FC < 0.67) to obtain that TRP is the most important amino acid for the patients after TIPS complicated with HE. Therefore, we further analyzed its metabolite levels in the PH patients after TIPS.

TRP is involved in various metabolic pathways in the body, and its metabolites play crucial roles in various diseases. Elevated serum TRP can promote the synthesis of 5-hydroxytryptophan (5-HTP). Studies have shown that an increase in 5-HTP is one of the important pathogenic mechanisms of HE (Jiang et al., 2018); however, in our study, we did not observe this change. Instead, we found higher levels of kynurenine and 2,3-pyridinedicarboxylic acid in the serum of patients with PH complicated with HE after TIPS than those without HE complications. We speculate that the occurrence of HE may be related to the overactivation of the kynurenine/IDO pathway of TRP metabolism in these patients.

Previous studies have demonstrated that pro-inflammatory cytokines can upregulate the expression of IDO under inflammatory conditions, thereby activating the KYN metabolic pathway and metabolizing KYN into neurotoxic metabolite quinoline acid (2,3-pyridinedicarboxylic acid), which is consistent with our results. Basile revealed high levels of quinoline acid in the brain and serum of animals with HE, establishing an association between HE and the KYN/IDO pathway (Basile et al., 1995). In the future, we plan to conduct systematic *in vivo* experiments to verify the relationship between the incidence of HE after TIPS and the activated kynurenine/IDO pathway.

To our knowledge, the occurrence of HE is closely related to elevated blood ammonia levels. However, as shown in the clinical information on patients in Table 1, there is no significant difference in blood ammonia between the two groups of patients. It is likely because the serum sample in our study is collected from the preoperative jugular vein blood, indicating that it is not feasible to predict the occurrence of postoperative HE based on blood ammonia levels before TIPS. Although the level of blood ammonia does not increase, the kynurenine/IDO pathway has already been activated in some patients before TIPS due to other physiological mechanisms. Therefore, we believed that the results of our study can predict the occurrence of postoperative HE by detecting TRP/KYN/QA levels before TIPS, which can help clinicians facilitate clinical decision-making strategies.

## 5 Conclusion

In summary, this study analyzed the 20 amino acids and 13 tryptophan metabolites in the preoperative serum of patients with/without HE after TIPS surgery using the targeted metabolomics. The results showed a strong correlation between serum TRP and the occurrence of HE after TIPS. Furthermore, TRP metabolite analysis revealed that higher levels of serum kynurenine and 2,3-pyridinedicarboxylic acid were found in the patients after TIPS complicated with HE, which may be due to the abnormal activity of the kynurenine/IDO pathway in their bodies. ROC multivariate analysis found that these three metabolites (TRP, KYN, and QA) as a combined form had better predictive value in predicting whether HE occurs in PH patients who underwent TIPS. However, the sample size included was relatively small, and the correlation between tryptophan and its metabolites and HE grading could not be further analyzed. In the future, the sample size will be further expanded to verify the results of this study, and the relationship between metabolites and HE grading will be analyzed in depth.

## Data Availability

The raw data supporting the conclusions of this article will be made available by the authors, without undue reservation.
