# Supplementary material for: Analysis of serum amino acids and tryptophan metabolites to predict hepatic encephalopathy in portal hypertension patients receiving a transjugular intrahepatic portal shunt (TIPS)
Source: Front Pharmacol. 2025 Aug 28;16:1546665. doi: 10.3389/fphar.2025.1546665 (PMC12423102; doi:10.3389/fphar.2025.1546665)
Supplement: Supplementary file 1 [file DataSheet1.docx]

Supplementary Material

Analysis of serum amino acids and tryptophan metabolites in patients with portal hypertension complicated with hepatic encephalopathy after transjugular intrahepatic portal shunt

Li Bao^1,2,*^, Yifan Lv^3^, Chunjing Yang^1,2^, Jingfeng Li^1,2^, Zhengyuan Shi^1,2^, Fuquan Liu^3^

^1^Department of Pharmacy, Beijing Shijitan Hospital, Capital Medical University, No.10 Tieyi-Road, Haidian District, Beijing, China;

^2^Beijing Key Laboratory of Bio-characteristic Profiling for Evaluation of Rational Drug Use, No.10 Tieyi-Road, Haidian District, Beijing, China;

^3^Department of Interventional Therapy, Beijing Shijitan Hospital, Capital Medical University, No.10 Tieyi-Road, Haidian District, Beijing, China.

*** Correspondence:**Li Bao
baoli3712@bjsjth.cn

# Supplementary Figures and Tables

## Supplementary Figures


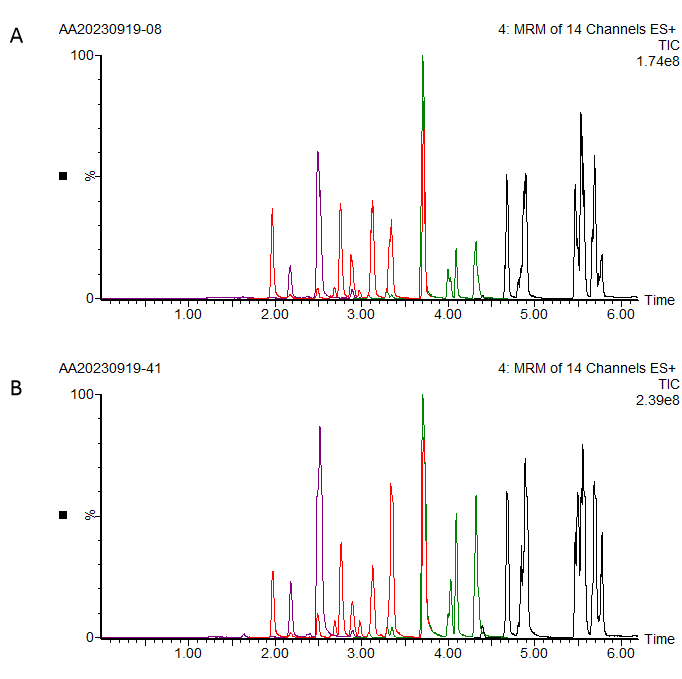


**Supplementary Figure 1.** Representative MRM chromatography from amino acid quantification of PH patients with (A) /without (B) HE after TIPS


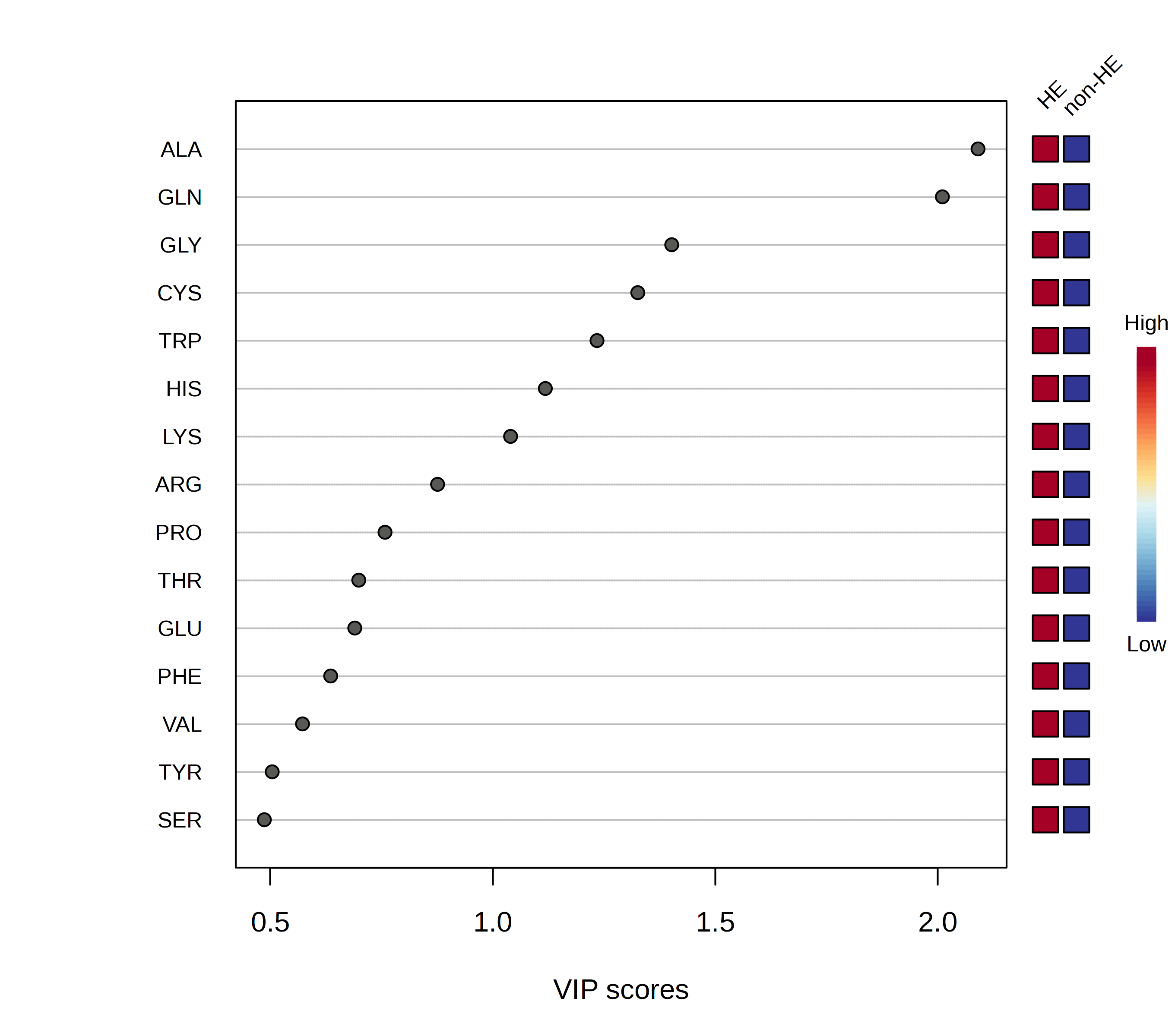


**Supplementary Figure 2.** VIP scores represent the most contributing amino acids involved in the separation between PH patients with (A) /without (B) HE after TIPS


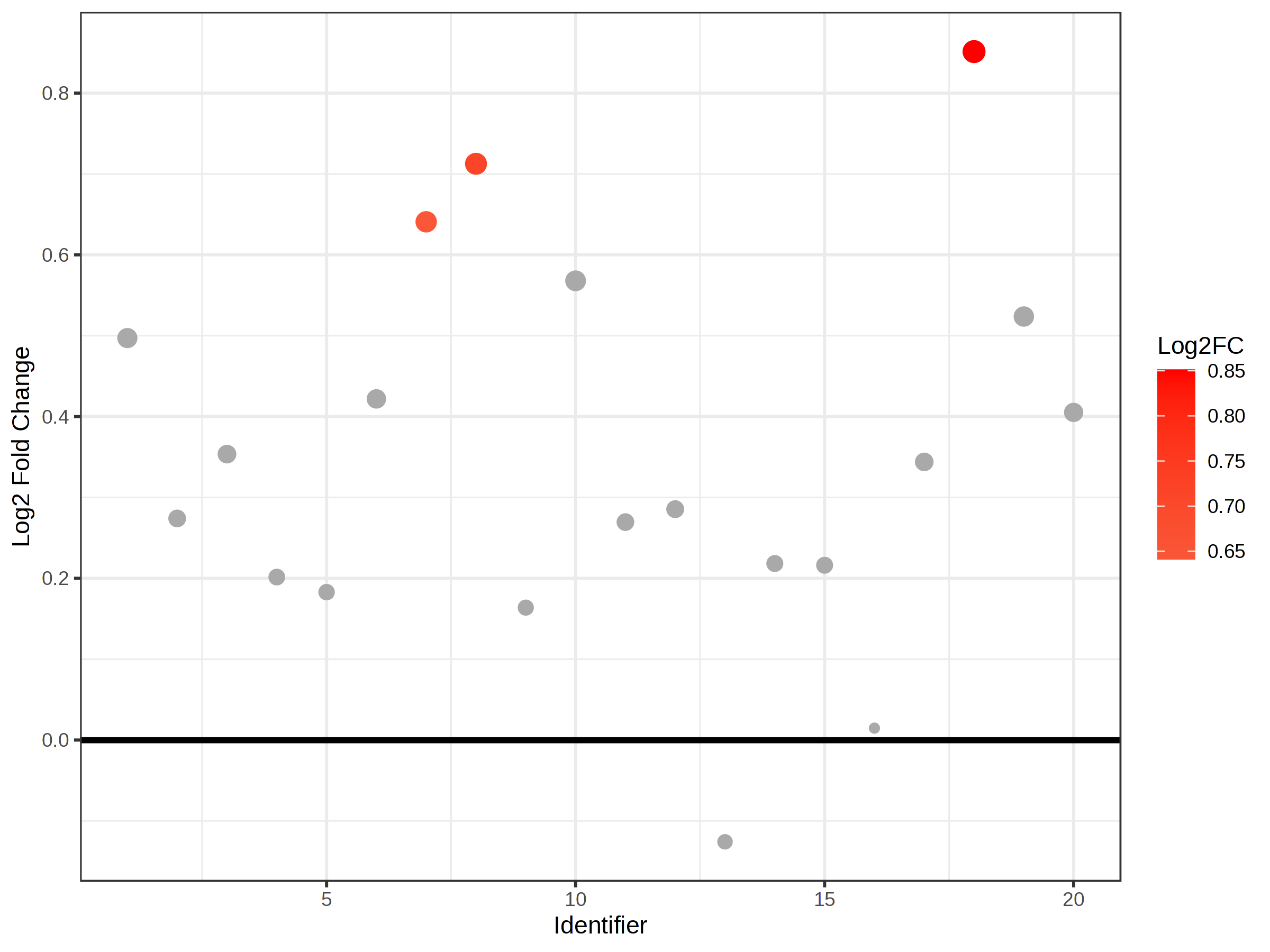


**Supplementary Figure 3.** Fold change (FC) of the significantly changed amino acids in PH patients with HE after TIPS (FC was set as >1.5)


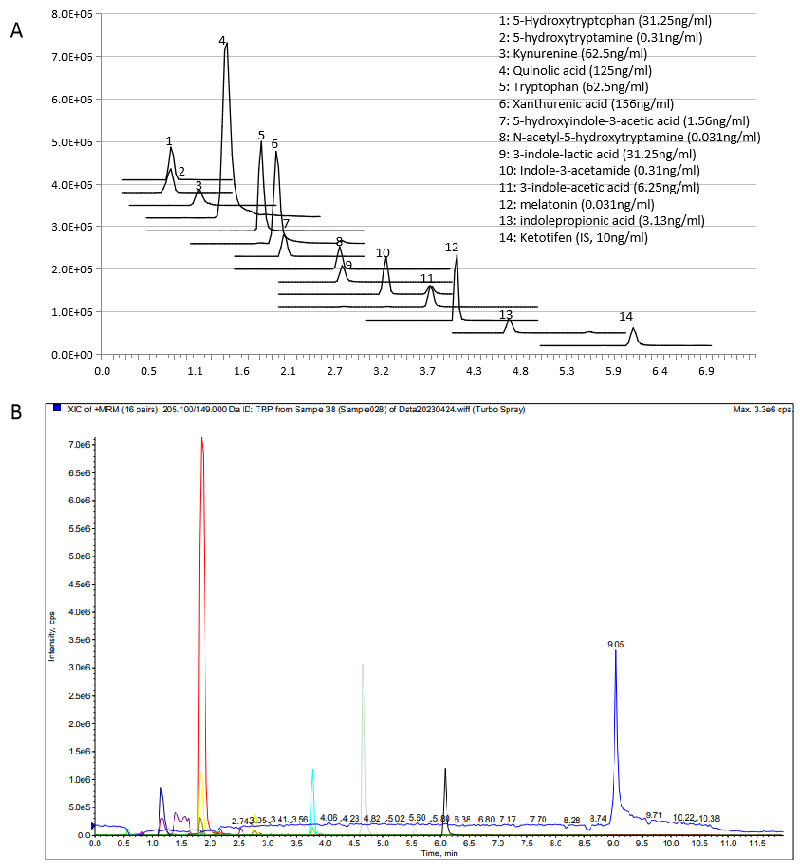


**Supplementary Figure 4.** (A)Chromatographic schematic sketch of 13 major tryptophan metabolites standards , (B) Chromatogram of human serum sample.

## Supplementary Tables

**Supplementary Table 1.** Standard curve linearity data of tryptophan metabolites obtained for the analyses

| Amino Acid | abbreviation | Linear equation | r | Linearity range |
| --- | --- | --- | --- | --- |
| Tryptophan | TRP | Y=0.00929X+0.03011 | 0.98379 | 10ng/ml-10μg/ml |
| Serotonin | 5-HT | Y=0.31620X+0.00890 | 0.98444 | 0.02ng/ml-10ng/ml |
| 5-hydroxytryptophan | 5-HTP | Y=0.02052X+0.03238 | 0.98251 | 1ng/ml-100ng/ml |
| N-acetyl-5-hydroxytryptamine | NAM | Y=3.32851e^-4^X+0.10881 | 0.99773 | 20pg/ml-1ng/ml |
| Melatonin | MT | Y=9.35099e^-4^X+0.00759 | 0.98944 | 1pg/ml-1ng/ml |
| 5-hydroxyindole-3-acetic acid | HIAA | Y=0.03706X+4.56197e^-4^ | 0.99842 | 0.2ng/ml-50ng/ml |
| 3-Indoleacetic acid | IAA | Y=0.03584X+0.00276 | 0.99404 | 0.2ng/ml-200ng/ml |
| 3-Indole-lactic acid | ILA | Y=0.00163X+0.00111 | 0.99708 | 1ng/ml-1μg/ml |
| Indolepropionic acid | IPA | Y=0.06503X+0.02331 | 0.98514 | 1ng/ml-10ng/ml |
| Indole-3-acetamide | IAM | Y=0.25551X+0.00782 | 0.99631 | 20pg/ml-10ng/m |
| Kynurenine | KYN | Y=0.00262X+8.73485e^-4^ | 0.99851 | 1ng/ml-2μg/ml |
| 2,3-Pyridinedicarboxylic acid | QA | Y=0.00779X+0.01927 | 0.98767 | 1ng/ml-200ng/ml |
| Xanthurenic acid | XA | Y=0.00316X+0.00574 | 0.99606 | 1ng/ml-2μg/ml |

**Supplementary Table** 2. Univariate and multivariate analysis of 3 differential metabolites

|  | univariate analyse | | | multivariate analysis | |
| --- | --- | --- | --- | --- | --- |
|  | AUC | OR (95% CI) | P value | AUC | OR (95% CI) |
| TRP | 0.888 | 0.771-0.963 | <0.001 | 0.916 | 0.812-1 |
| KYN | 0.787 | 0.646-0.899 | <0.001 |  |  |
| QA | 0.781 | 0.661-0.894 | 0.002 |  |  |
